# Asthma Trajectories in Early Childhood: Identifying Modifiable Factors

**DOI:** 10.1371/journal.pone.0111922

**Published:** 2014-11-07

**Authors:** Lidia Panico, Beth Stuart, Mel Bartley, Yvonne Kelly

**Affiliations:** 1 Institut National d'Etudes Démographiques, Paris, France; 2 Faculty of Medicine, University of Southampton, Southampton, United Kingdom; 3 International Centre for Lifecourse Studies, Department for Epidemiology and Population Health, University College London, London, United Kingdom; UNC School of Dentistry, University of North Carolina-Chapel Hill, United States of America

## Abstract

**Background:**

There are conflicting views as to whether childhood wheezing represents several discreet entities or a single but variable disease. Classification has centered on phenotypes often derived using subjective criteria, small samples, and/or with little data for young children. This is particularly problematic as asthmatic features appear to be entrenched by age 6/7. In this paper we aim to: identify longitudinal trajectories of wheeze and other atopic symptoms in early childhood; characterize the resulting trajectories by the socio-economic background of children; and identify potentially modifiable processes in infancy correlated with these trajectories.

**Data and Methods:**

The Millennium Cohort Study is a large, representative birth cohort of British children born in 2000–2002. Our analytical sample includes 11,632 children with data on key variables (wheeze in the last year; ever hay-fever and/or eczema) reported by the main carers at age 3, 5 and 7 using a validated tool, the International Study of Asthma and Allergies in Childhood module. We employ longitudinal Latent Class Analysis, a clustering methodology which identifies classes underlying the observed population heterogeneity.

**Results:**

Our model distinguished four latent trajectories: a trajectory with both low levels of wheeze and other atopic symptoms (54% of the sample); a trajectory with low levels of wheeze but high prevalence of other atopic symptoms (29%); a trajectory with high prevalence of both wheeze and other atopic symptoms (9%); and a trajectory with high levels of wheeze but low levels of other atopic symptoms (8%). These groups differed in terms of socio-economic markers and potential intervenable factors, including household damp and breastfeeding initiation.

**Conclusion:**

Using data-driven techniques, we derived four trajectories of asthmatic symptoms in early childhood in a large, population based sample. These groups differ in terms of their socio-economic profiles. We identified correlated intervenable pathways in infancy, including household damp and breastfeeding initiation.

## Introduction

In developed countries, both the incidence and the prevalence of asthma has increased dramatically in the last few decades [1], [2]. Asthma and wheezing illnesses are common in childhood, with about 1 in 5 British children reporting recent wheezing symptoms [3], [4] and a similar proportion reporting a doctor diagnosis of asthma [5]. Asthma is a heterogeneous disease with different clinical expressions and there is a conflicting view as to whether associated symptoms in childhood represent several discreet entities or a single but variable disease. Current classifications, such as those based on the Tucson Children's Respiratory Health Study of early transient, late onset and persistent wheeze [6], have become popular as a basis for academic research and are also increasingly used in clinical settings [7].

Studying asthma trajectories in childhood is important because different patterns of asthma expressed during childhood are important predictors of future outcomes [8], and because asthma trajectories appear to entrench early, possibly before age 6/7 [9]. Therefore, detecting early predictors of later childhood respiratory health may be important to identify interventions to potentially alter the course of the disease. However, little data is available for very young children, and therefore most studies investigating asthma phenotypes start with school aged children. Furthermore, many studies have been carried out in clinical rather than population-based samples and have relied on subjective classifications by researchers. This may explain why it has been difficult to replicate asthma groupings across studies, a key step to determine whether these groupings constitute “real” discrete phenotypes.

While studies have focused on describing clinical features, few studies have explicitly tested differences in socio-economic characteristics between asthma groupings. However, such analyses can help better understand the causation of asthma. Furthermore, when thinking about potential interventions, most studies have considered various aspects of clinical treatments, there has been less exploring potential intervenable factors in the child's environment, such as household damp and parental smoking, that could be of interest to public policy. Therefore, this paper aims to identify longitudinal trajectories of wheeze and other atopic symptoms, starting in early childhood, using a large, nationally representative sample of British children; we then describe the socio-economic profiles of these different groups of children, and we aim to identify potentially modifiable processes in infancy correlated with these different trajectories.

In this paper we propose to use a prospective birth cohort, the British Millennium Cohort Study (MCS), which follows over 19,000 children at regular intervals from 9 months after birth. As a large multi-purpose study, the MCS does not have the scope to make clinical measurements. However, the MCS does include a validated instrument to measure asthma and wheezing symptoms, the International Study of Asthma and Allergies in Childhood module, as well as detailed information on the socio-economic background and the home environment. Such variables are often measured out as “confounders” in asthma research. Therefore, this study does not aim to add to the already rich literature on the clinical features of asthma phenotypes. Instead, using a large, population-based sample, we aim to identify and characterize groups of children according to their reported symptoms in early childhood using data driven clustering methodologies, and, crucially, to identify early environmental and potentially intervenable influences on later respiratory health.

## Context

### The importance of early childhood for the genesis of asthma

Deficits in lung function, bronchial hyper-responsiveness, and structural airway remodeling are key features of asthma, and they appear to be established during the pre-school years in most individuals [9]. For example, the population-based Melbourne Asthma Study, which followed 401 children from age 7 in 1964 [8], found that no significant loss of pulmonary function occurred after ages 7–10, even in individuals with a severe disease at age 35. More recently, data from a New Zealand cohort of about 1000 children followed from age 9 also showed that lower levels of lung function in patients with persistent asthma were already evident by the start of the study with no further deterioration after that age [10]. The Tucson Children's Respiratory Study, a cohort of 1,246 newborns born between 1980 and 1984, showed that, for children with persistent wheezing symptoms, lung function deficits were not related to poorer lung function shortly after birth, but such deficits were already evident by age 6 and probably irreversible by age 9 [6]. This highlights the importance of the early years for the emergence and the entrenchment of life-long asthma trajectories.

### Associations with socio-economic characteristics and intevenable factors

Although the association of lung function and other clinical outcomes with asthma phenotypes are well documented, the variation in environmental risk factors across asthma groups has been less explored. We know that there is a cross sectional association between socio-economic position and current asthma or lung function in adults [11]–[14], and a smaller body of work has found similar associations in childhood [15]–[17]. How these associations vary across longitudinal measures of asthma has been less explicitly explored as such factors are often considered as confounders in that particular literature.

On the other hand, we know that there are strong associations between asthma and a number of potentially modifiable factors such as exposure to smoking. Such exposures are also often controlled out as “confounders” [18]. Exceptions include studies that have shown that the “transient early phenotype” (children who wheeze in early childhood whose symptoms resolve by school age) appears to be linked to prematurity, having siblings, attending group care settings, maternal smoking during pregnancy and post-natal exposure to smoke [19]–[23]. Daycare attendance appears to increase the risk of early wheeze but protect against older childhood wheezing [23], [24].

### Classifications derivation

Asthma classifications are useful both for research and to assist in clinical diagnosis. While the classifications derived in the literature have provided useful descriptions, they have been difficult to replicate across datasets, a key to establish whether the suggested categorizations are indeed true disease phenotypes. Currently, there is a wide range of concepts among clinicians [25] and no phenotype model appears to be more valid than others [26], with perhaps the exception of the “early transient wheeze” group which has been identified by different studies [27]. Some of these problems may exist because models do not allow multiple features or complex group definitions. Furthermore, the samples used (clinical or community populations) may be problematic. Small sample sizes may compound the problem.

Classifications are often subjective, using criteria suggested by clinicians or researchers, such as physiological characteristics (atopy, severity, age at onset, chronic airflow obstruction), triggers (exercise, allergens, irritants, viral infection) or their time course (transient, early onset, late onset, persistent). Classic categorizations derived in this manner include the Tucson Children's Respiratory Study's groupings. Martinez and colleagues [6] proposed a four category phenotype: non-wheezers, transient wheezers (at least one wheezing symptom before age 3, no wheezing at 6 years), late onset wheezers (no wheeze until age 3, wheezing at 6 years), and persistent wheeze (wheezed in the first 3 years of life, wheezing at age 6). The last group is sometime split into non-atopic persistent wheezers and atopic persistent wheezers [22], [28]. Using data from the New Zealand cohort, Sears and colleagues (2003) proposed six longitudinal wheezing phenotypes: persistent wheeze, relapse (wheeze stopped then recurred), in remission, intermittent wheezing, transient wheezing, and wheezing never reported.

This subjective approach produces easily understood classifications, which can be easily applied in both research and clinical practice. However, rather than allow the researcher to subjectively determine the classifications, it is possible to use data-driven techniques to explore whether homogeneous subgroups exist within the study population. These techniques have recently been explored in the context of childhood asthma trajectories by two papers using a sample from the Avon Longitudinal Study of Parents and Children (ALSPAC), a community based sample of children born in Avon, a British county. Henderson and colleagues (2008) used a longitudinal LCA model applied to reports of wheezing from 5,760 children at several time points between ages 1 and 8 and identified 5 wheezing groups: never/infrequent wheeze (75%), intermediate onset wheeze (3%), persistent wheeze (3.5%), transient early wheeze (17%), and late onset wheeze (2%). The same model was tested on the Dutch PIAMA (Prevention and Incidence of Asthma and Mite Allergy) study [29]. Based on a sample of about 2,800 children, similar classifications were derived. However, both studies were relatively small and not nationally representative. As far as we are aware, no study has been able to use a large, nationally representative sample to derive asthma trajectories.

## Research Aims

We have three main research aims that structure our analyses: (1) to identify longitudinal trajectories of wheeze and atopic symptoms in early childhood using longitudinal latent class analysis in a large, nationally representative birth cohort; (2) to characterize the resulting trajectories according to the socio-economic background of children and their households at 9 months of age; and (3) to use multinomial regression techniques to identify modifiable processes at 9 months correlated with these trajectories, taking into account socio-economic profiles.

## Data and Methods

### The Millennium Cohort Study

The Millennium Cohort Study (MCS) is a nationally representative birth cohort study of infants born in the UK from September 2000 to January 2002. The survey design, recruitment process and fieldwork have been described in detail elsewhere [30]. Briefly, 18553 households agreed to participate in the initial survey, an overall response rate of 68%. Households were identified through the Department of Work and Pensions Child Benefit system, and selected on the basis of where the family was resident shortly after the time of birth. Uptake of Child Benefit is almost universal (98%). The sample has a probability design and is clustered at the electoral ward level, with disadvantaged residential areas and areas with a high proportion of ethnic minority population being over represented.

We use data from the first wave of data collection carried out when the child was about 9 months of age to characterize the socio-economic profiles of the households and identify potentially intervenable factors. For classification purposes, we use data from the second, third and fourth wave of interviews, carried out through home visits when the cohort member was aged approximately 3, 5 and 7 years, respectively. Data was collected through home interviews with the main carer, usually the mother. The overall sample size for wave 2 was 15 307, 15 246 at wave 3, and 14 043 at wave 4. This analysis is based on 11,632 cases with complete data on questions on recent wheeze, and ever eczema and ever hayfever at ages 3, 5 and 7.

### Variables employed

At ages 3, 5 and 7, the ISAAC (International Study of Asthma and Allergies in Childhood) core questionnaire for asthma was included in the interview with the main carer. ISAAC is a widely used and validated instrument to measure childhood asthma and wheezing illnesses and includes questions on the occurrence of asthma and wheezing symptoms [31] (see Annex S1). For the latent class analysis, we use the question on recent wheeze (reported as whether the child wheezed in the last year) at ages 3, 5 and 7. The second key variable used is a proxy for atopy, namely other atopic symptoms. Studies often ignore the distinction between atopic and non-atopic asthma even though these phenotypes are likely to have distinct causal mechanisms [32]. As atopy cannot be measured objectively in the MCS, we rely on two questions on other atopic symptoms (hayfever and eczema), as reported by the parent. Questions asked to the main carer at ages 3, 5 and 7 on whether the child ever had eczema and whether the child ever had hay fever, adapted from the ISAAC module, are used. In this proxy therefore atopic simply means the absence of eczema and/or hay fever, and may include some asymptomatic children who are atopic (by skin test or IgE levels). Cases with complete information at all waves on these key variables were retained (n = 11,632).

A number of markers for socio-economic background were considered. We used a forward selection exercise, a data-driven model building approach, to reduce the number of variables included in the model. Under this approach, variables are added to the model one at a time and tested for inclusion. The most significant variables are retained in the model, as long as its p-value is below a pre-set level. It is customary to set p-values above the conventional.05 level because of the exploratory nature of this method. The exercise begins with the variable that appears to be most significant in initial analyses, and continue adding variables until none of remaining variables are "significant" when added to the model. Following this exercise, we retained three socio-economic variables which were significant at the level p = 0.20: parental income, parental education, and a persistent poverty indicator. This allowed us to keep the model relatively simple while maximising its predictive power.

The first two variables are based on the first interview with the main carer, carried out when the child was about 9 months old. Annual parental income is modelled as a log-transformed measure; and parental education is operationalised by the highest educational qualification for either resident parents (no educational qualifications; only overseas qualifications; qualifications equivalent to an NVQ1, NVQ2, NVQ3, NVQ4 and NVQ5). The National Vocational Qualifications (NVQs) is a system of competence-based education and training that aims to recognize individual levels of competence. The framework indicates the “equivalence” of both vocational and academic qualifications, and reflects the level of skills acquired. As an indicator, an NVQ5 is equivalent to a graduate degree; an NVQ3 is equivalent to two A-levels, a high-school qualification. A persistent poverty indicator, which captures the frequency the household was classed as poor over the 4 waves of data collection (from 9 months to 7 years of age), was calculated using data from those waves. Households were classed as poor if their equivalised income was 60% below the mean income for that wave. Equivalised income takes into account household composition and was calculated using the McClements equivalence scale [33].

To explore potentially intervenable processes, a number of variables were selected, measured at the 9 month interview. All variables are binary (yes/no) unless otherwise indicated. Overcrowding was defined as having more than one individual per room, excluding the bathroom and kitchen; damp in the home is measured on a five point scale ranging from no problems with damp to severe damp problems (modelled as a continuous variable); exposure to tobacco smoke was defined as whether either resident parental figure smokes; any smoking during pregnancy by the mother is also included; breastfeeding initiation, irrespective of duration, was included. A number of potential confounders linked to an increased risk of asthma and wheezing symptoms were included in the analytical models: the child's gender; whether the child weighed less than 2500 grams at birth; and the number of co-resident siblings in infancy (modelled as a continuous variable).

### Statistical analysis

For classification purposes, we use longitudinal Latent Class Analysis (LLCA), a data driven approach to derive categories. Using the sequences of responses at each wave for each child, LLCA identifies groups that share similar longitudinal response patterns. It assumes that the population is composed of subsets (latent classes), each having distinctive distributions of the key variables [34]. The LLCA model estimates two sets of parameters: the conditional response probabilities and the latent class prevalences. The conditional response probabilities give the probability of observing a particular response pattern within the group. So, for example, the probability that an individual in latent class 1 will respond “Yes” at age 3 to “Has your child wheezed in the last year?”. The latent class prevalence gives the proportion of children in each latent class.

In order to choose the appropriate number of latent classes, the model was repeatedly fitted with the number of latent classes increasing step-wise from 1 to 6. The calculations were carried out in LEM, a statistical package designed specifically for categorical data analysis, including latent class models, with parameters estimated using maximum likelihood criterion, computed using the Expectation-maximization (EM) algorithm [35]. There is no single statistical test to determine the correct number of latent classes [36]. Selecting the best model requires a consideration both of the substantive interpretation of the output and the statistical measures of model fit. We assessed model fit using a range of measures: the Bayesian Information Criteria (BIC), the dissimilarity index, the likelihood ratio chi-squared test statistic, and the percentage of classification error based on modal assignment [37], [38].

The model allocates individuals to the latent class for which they have the highest probability, called modal allocation. Sensitivity analyses were carried out using a random allocation, which classified differently cases were individuals have similar probabilities of belonging to two different classes. These analyses confirmed the substantive findings presented below.

To evaluate the distribution of the latent classes with respect to key covariates we use multinomial logistic regression carried out in Stata 11 [39]. These models produce Relative Risk Ratios (RRRs) which estimate the relative risk of a child belonging to a given class compared to the reference class, which is always the largest group. We first assessed the relative importance of each intervenable factor (household damp, breastfeeding initiation, parental smoke, and overcrowding) individually, taking into account a number of socio-economic markers and confounding factors. We then test a full model, which includes all intervenable factors alongside the full set of socio-economic and control variables. Survey weights are applied to all cross tabulations and regression analyses. This allows correcting for cohort members having unequal probabilities of selection in the study due to the stratified and clustered sample design, as well as to take account of attrition between data collection waves and unit non-response [40], [41].

## Results

### Descriptive sample characteristics

Weighted analyses showed that, by age 7, 15.1% of children reported ever having had asthma and 25.8% had ever wheezed. 42.9% reported ever having eczema or hay fever (see Table 1). Reports of recent wheeze were higher in early childhood (at age 3, 18.5% of children had wheezed in the previous 12 months) than in the primary school years (at age 7, 11.4% of children reported wheezing in the previous 12 months). The rest of the results section is structured around the three main aims of this paper: (1) to identify longitudinal latent trajectories of wheeze and other atopic symptoms; (2) to characterize the resulting trajectories by a number of household socio-economic variables measured in infancy; and (3) to identify modifiable processes in infancy which may be correlated with these trajectories, taking into account differing socio-economic profiles.

**Table 1:** Prevalence of asthma and wheezing symptoms at ages 3, 5 & 7.

	Age 3 %	Age 5 %	Age 7 %
**Ever had asthma**	11.5	13.7	15.1
**Ever had wheeze**	29.3	28.4	25.8
**Wheezed in last year**	18.5	15.0	11.4
**Ever had eczema and/or hay fever**	37.8	40.1	42.9
**Total sample size**	11,632		

Weighted percentages.

### Aim 1: Latent trajectories identification

For this aim, we use our key variables (wheeze, and the atopy proxy, namely other atopic symptoms) to derive latent trajectories of symptoms. We tested a number of models with different number of classes, Table 2 gives the measures of model fit that we used to select the solution with the optimal number of classes. The four class solution was chosen as the best fit as it had the lowest BIC value, a dissimilarity index below 0.05, a significant p-value on the chi-squared LRT and an acceptable level of potential classification error (9% of children).

**Table 2:** Measures of model fit, Longitudinal Latent Class model of wheeze and other atopic symptoms.

Number of latent classes	BIC	Dissimilarity index	Likelihood ratio chi-squared test statistic	Percentage classification error based on modal class assignment
**1**	76455.00	0.4476	N/A	N/A
**2**	65549.56	0.1459	<0.0001	3.68%
**3**	63768.25	0.0644	<0.0001	5.86%
**4**	63029.01	0.0158	<0.0001	9.02%
**5**	63064.27	0.0138	<0.0001	19.60%
**6**	63118.96	0.0106	0.1462	59.42%

**Figure 1:**
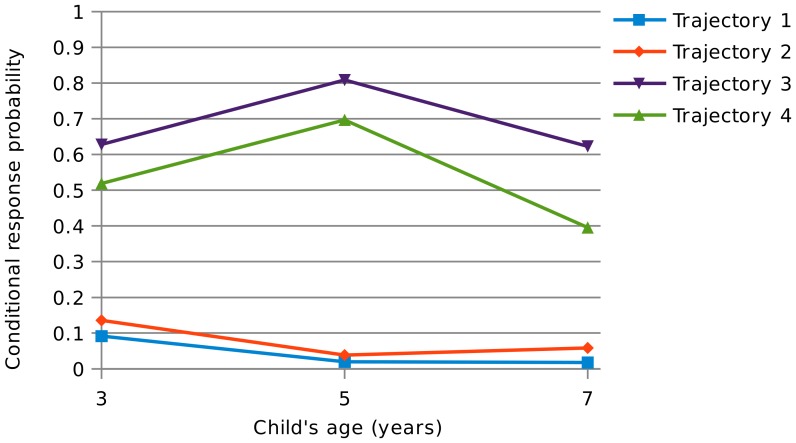
Conditional response probabilities of reporting wheeze in the last 12 months, ages 3 to 7. Trajectory 1: low levels of wheeze and low levels of other atopic symptoms; Trajectory 2: low levels of wheeze and high prevalence of other atopic symptoms; Trajectory 3: high levels of wheeze and high levels of other atopic symptoms; Trajectory 4 high levels of wheeze and low levels of other atopic symptoms.

**Figure 2:**
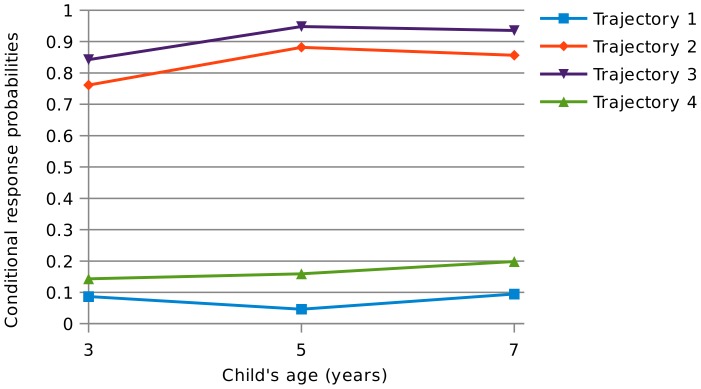
Conditional response probabilities of reporting ever eczema and/or ever hayfever, ages 3 to 7. Trajectory 1: low levels of wheeze and low levels of other atopic symptoms; Trajectory 2: low levels of wheeze and high prevalence of other atopic symptoms; Trajectory 3: high levels of wheeze and high levels of other atopic symptoms; Trajectory 4 high levels of wheeze and low levels of other atopic symptoms.

Table 3 shows the categorizations produced by the four class model. The four identified latent trajectories of wheeze and other atopic symptoms from age 3 to 7 emerging from this model are also shown on Figures 1 and 2. They can be summarized as follows:

**Table 3:** Conditional response probabilities for the key model variables (recent wheeze, and other atopic symptoms) by latent trajectories (1).

	Trajectory 1	Trajectory 2	Trajectory 3	Trajectory 4
**Recent wheeze, age 3**	0.0913	0.1358	0.6279	0.5190
**Recent wheeze, age 5**	0.0195	0.0385	0.8088	0.6968
**Recent wheeze, age 7**	0.0176	0.0583	0.6231	0.3958
**Ever eczema and/or hayfever, age 3**	0.0868	0.7611	0.8427	0.1432
**Ever eczema and/or hayfever, age 5**	0.0461	0.8821	0.9480	0.1592
**Ever eczema and/or hayfever, age 7**	0.0949	0.8564	0.9356	0.1986
**% of total sample (sample size)**	54% (6,275)	29% (3,393)	9% (1,043)	8% (921)

(1) Trajectory 1: low levels of wheeze and low levels of other atopic symptoms; Trajectory 2: low levels of wheeze and high prevalence of other atopic symptoms; Trajectory 3: high levels of wheeze and high levels of other atopic symptoms; Trajectory 4: high levels of wheeze and low levels of other atopic symptoms.

- Trajectory 1: a trajectory with both low levels of wheeze and other atopic symptoms (54% of children): this category is characterized by low levels of all symptoms. Children in this group who do wheeze are least likely to report severe or limiting symptoms (results available upon request).
- Trajectory 2: a trajectory with low levels of wheeze and high prevalence of other atopic symptoms (29% of children): this category has low levels of wheeze at all ages, however this group is characterized by high levels of eczema and/or hayfever, with about 85% of the sample reporting eczema or hay fever by age 7.
- Trajectory 3: a trajectory with a high prevalence of both wheeze and other atopic symptoms (9% of children): the “classic” asthmatic group, this category has the highest levels of wheeze (81% of the sample reported recent wheeze at age 5) and the highest proportion of children reporting eczema and/or hayfever, peaking at about 93% by age 7. Children in this group report the most severe wheezing symptoms and the most limitations due to wheeze.
- Trajectory 4: a trajectory with high levels of wheeze but low levels of other atopic symptoms (8% of children): this group had relatively high levels of wheeze, although both overall levels and severity of symptoms are below that of trajectory 3. Only about 20% of the sample reported eczema or hay fever by age 7 in this group.

### Aim 2: The socio-economic characteristics of the latent trajectories of wheeze and other atopic symptoms

Next, we describe the socio-economic characteristics of the four identified latent trajectories. Table 4 shows the characteristics of the groups in terms of their socio-economic profiles at the first wave of data available, when the children were aged about 9 months. Across a number of indicators, including parental income and education, trajectory 2 (low wheeze, high levels of other atopic symptoms) appears to be the most advantaged group, slightly more so than largest group, trajectory 1 (both low wheeze and other atopic symptoms). Trajectory 4 (high wheeze, low levels of other atopic symptoms) appears to be disproportionately disadvantaged. These differences extend to other early markers of well-being (see the second panel of Table 4): trajectory 2 was on average least likely to be born at a low birthweight, while trajectory 4 appeared to have the highest risk of being low weight at birth. Interestingly, if we compare each group with high levels of wheeze with its low-wheeze counterpart (i.e. trajectory 2 versus trajectory 3), it appears that the most advantaged of the two groups have the lower levels of wheeze.

**Table 4:** Socio-economic variables and exposure variables, by latent trajectories (1).

		Trajectory 1	Trajectory 2	Trajectory 3	Trajectory 4
**Socio-economic variables**					
Parental income, 9 months	Don't know	6.1	6.2	6.6	5.8
	Under 10 400 pounds	14.7	13.1	19.4	20.0
	10 400–20 800	27.8	28.5	30.4	32.4
	20 800–31 200	24.5	22.7	21.2	18.0
	31 200–52 000	19.1	20.8	16.3	18.3
	52 000 and over	7.7	8.6	6.1	5.5
	p-value	<0.001			
	Mean income, pounds	26 608	27 792	24 205	23 570
Highest parental education, 9 months	None of these	5.5	3.7	5.1	8.3
	Overseas quals only	1.2	1.0	1.7	1.7
	NVQ 1	4.3	3.8	5.0	7.5
	NVQ 2	22.8	21.5	24.9	23.9
	NVQ 3	16.7	15.6	14.3	17.3
	NVQ 4	41.3	45.7	42.1	35.1
	NVQ 5	8.2	8.7	7.0	6.3
	p-value	<0.001			
Persistent poverty indicator, 9 months to 7 years	Never poor	64.7	67.3	58.4	54.7
	Poor at one wave	12.9	14.2	13.2	16.2
	Poor at two waves	7.0	6.6	8.7	9.3
	Poor at three waves	7.1	5.3	9.6	8.7
	Always poor	8.3	6.6	10.0	11.0
	p-value	<0.001			
**Exposures**					
Overcrowding	% overcrowded home	10.9	7.5	9.6	12.4
Either parent smokes	% parental smoke	41.6	39.9	44.3	48.8
Damp home	Yes	11.8	14.7	14.7	16.1
Breastfeeding initiated	Yes	71.1	74.2	69.7	64.2
**Confounders**					
Gender	Male	48.1	49.7	62.1	56.3
	p-value	<0.001			
Low birthweight	% low birthweight	6.7	5.0	7.7	9.8
	p-value	0.002			
Formal group care	% formal group care	6.5	6.6	6.9	4.7
	p-value	0.0501			

Weighted percentages.

(1) Trajectory 1: low levels of wheeze and low levels of other atopic symptoms; Trajectory 2: low levels of wheeze and high prevalence of other atopic symptoms; Trajectory 3: high levels of wheeze and high levels of other atopic symptoms; Trajectory 4: high levels of wheeze and low levels of other atopic symptoms.

The third panel of Table 4 shows that when looking at characteristics normally associated with atopy such as gender and sibship size, the groups perform as expected: the two groups reporting high levels of eczema and/or hayfever are more likely to include boys than the groups with low levels of these symptoms, and are more likely not to have older siblings. There was however no clear associations with being exposed to group care at a young age.

### Aim 3: Identifying intervenable factors

The final phase of analyses consisted of identifying potentially intervenable predictors, taking account of the differing socio-economic profiles and a number of control factors such as sex and sibship size. Table 5 shows the multinomial logistic regressions for the relative risk of being in a given trajectory compared to being in the largest trajectory. The results suggest that, once their more deprived socio-economic background is accounted for, the children in trajectory 4 were more likely to have been exposed to household damp at 9 months of age, and were less likely to have been breastfed. In the fully adjusted model, when all potentially intervenable factors are included, breastfeeding initiation and household damp remain significant. Both groups reporting high levels of eczema and/or hayfever (trajectories 2 and 3) were more likely to be exposed to household damp at 9 months in the fully adjusted model.

**Table 5:** Multinomial logistic regression of intervenable factors by latent trajectories (1).

	Model 1	Model 2	Model 3	Model 4	Model 5	Model 6
	RRR	RRR	RRR	RRR	RRR	RRR
**Trajectory 1**	— reference class —					
**Trajectory 2**						
Breastfeeding initiation	0.94					0.92
Parental smoke, 9 months		0.98				0.99
Smoke during pregnancy			0.96			0.95
Damp, 9 months				1.31*		1.35*
Overcrowd, 9 months					0.95	.94
Log income, 9 months	.86	.86*	.87	.87	.86*	.89
No educational quals	.68	.68	.70	.69	.68	.70
Overseas ed quals only	.91	.91	.92	.89	.91	.91
NVQ 1	.84	.85	.84	.83	.85	.82
NVQ 2	.76*	.76*	.76	.75*	.76*	.75*
NVQ 4	1.11	1.10	1.10	1.10	1.10	1.11
NVQ 5	1.00	0.99	0.98	0.99	1.00	0.98
Poor at one wave	1.10	1.11	1.13	1.10	1.10	1.12
Poor at two waves	1.31	1.33	1.31	1.38	1.33	1.27
Poor at three waves	1.52*	1.53*	1.58*	1.51*	1.53*	1.57*
Always poor	1.35	1.37	1.43	1.32	1.37	1.40
Gender	.54***	.54***	.54***	.54***	.54***	.54***
Low birth weight	.94	.94	.94	.93	.94	.93
Co-resident siblings	.84**	.84**	.84**	.84**	.84**	.84**
**Trajectory 3**						
Breastfeeding initiation	1.04					1.03
Parental smoke, 9 months		0.98				1.02
Smoke during pregnancy			0.91			.89
Damp, 9 months				1.25*		1.26*
Overcrowd, 9 months					0.91	.88
Income, 9 months	0.97	0.97	0.97	0.98	0.97	0.98
No educational quals	.88	.87	.88	.87	0.87	.88
Overseas ed quals only	.82	.82	.86	.82	0.82	.82
NVQ 1	1.07	1.06	1.06	1.06	1.07	1.06
NVQ 2	0.91	0.92	0.90	0.92	0.92	0.90
NVQ 4	1.10	1.10	1.09	1.10	1.10	1.08
NVQ 5	0.99	1.00	0.99	1.01	1.01	0.98
Poor at one wave	1.10	1.11	1.13	1.10	1.11	1.13
Poor at two waves	.96	.96	.98	.94	.96	.96
Poor at three waves	.79	.80	.82	.78	.80	.81
Always poor	.88	.88	.92	.86	.89	.91
Gender	.96	.96	.96	.96	.96	.96
Low birth weight	1.33*	1.33*	1.31*	1.33*	1.33*	1.30*
Co-resident siblings	.84***	.84***	.84***	.84***	.85***	.85***
**Trajectory 4**						
Breastfeeding initiation	.79*					.78*
Parental smoke, 9 months		1.17				1.10
Smoke during pregnancy			1.22			1.36
Damp, 9 months				1.38*		1.36*
Overcrowd, 9 months					1.34	1.32
Income, 9 months	.90	.89	.90	.90	.89	.92
No educational quals	1.33	1.38	1.33	1.38	1.37	1.27
Overseas ed quals only	1.18	1.16	0.95	1.13	1.13	0.94
NVQ 1	1.58*	1.62*	1.56*	1.60*	1.62*	1.49*
NVQ 2	1.02	1.02	1.02	1.00	1.01	1.03
NVQ 4	.93	.91	.92	.89	0.89	.97
NVQ 5	.96	.94	.94	.90	0.92	1.02
Poor at one wave	1.26	1.26	1.28	1.26	1.26	1.26
Poor at two waves	1.24	1.23	1.22	1.21	1.24	1.14
Poor at three waves	1.02	1.00	0.99	1.01	1.01	0.96
Always poor	1.01	0.99	0.97	0.99	0.98	0.90
Gender	.74**	.74**	.77**	.75**	.75**	.75**
Low birth weight	.75	.76	.73	.75	.76	.74
Co-resident siblings	1.03	1.05	1.04	1.04	1.02	1.00

Weighted Relative Risk Ratios.

*** p<0,001** p <0,01 *p<0,05.

(1) Trajectory 1: low levels of wheeze and low levels of other atopic symptoms; Trajectory 2: low levels of wheeze and high prevalence of other atopic symptoms; Trajectory 3: high levels of wheeze and high levels of other atopic symptoms; Trajectory 4: high levels of wheeze and low levels of other atopic symptoms.

## Discussion

In this work, we use parental reports of wheeze, eczema and hayfever at age 3, 5 and 7 from a large birth cohort representative of British children born in 2000–2001. We identify four latent trajectories of symptoms, and quantified their associations with a number of factors in infancy that could form a basis for intervention. We employed longitudinal Latent Class Analysis to derive categories in a data-driven manner based on the heterogeneity of our sample.

Our model distinguished four classes of children: (1) a trajectory with both low levels of wheeze and other atopic symptoms, into which over half our population is classed; (2) a trajectory with low levels of wheeze and the highest prevalence of other atopic symptoms, which included nearly a third of the population; (3) a trajectory with the highest prevalence of wheeze, and higher than average levels of other atopic symptoms, which comprised 9% of our sample; and (4) a trajectory with high levels of wheeze but low levels of other atopic symptoms, which included 8% of our population.

While a non-atopic high-wheeze group was identified in previous literature [42], [43], our identified trajectories differ from previously reported patterns of early childhood wheezing. Notably, they contrast with previous classifications that focus on early versus late onset of wheezing symptoms as one of the most important distinction. This dichotomy did not appear in our findings. Our results also challenge the current model that early wheezing is normally not associated with atopy, while late wheeze is associated with atopic symptoms. Such categorizations did not emerge from our data.

The main aim of this paper was to characterize the resulting latent trajectories according to the child's socio-economic background and to identify potentially intervenable factors in children's early environment. We found that the four groups were different in terms of a number of socio-economic markers. The group with low levels of wheeze but the highest levels of eczema and/or hayfever appeared to be the most advantaged in terms of parental incomes and educational qualifications, showing a slightly more advantaged profile than all other groups. The two groups with high levels of wheeze were less advantaged. The group with high levels of wheeze but low levels of other atopic symptoms appeared to be the most disadvantaged, showing the lowest incomes and fewest parental educational qualifications. Taking into account these differing socio-economic backgrounds, we looked at a number of potential modifiable factors. In fully adjusted models, the two groups with high levels of eczema and/or hayfever were more likely to have lived in damp homes in infancy than the largest group. The trajectory reporting high levels of wheeze but low levels of other atopic symptoms appeared to be less likely to have been breastfed and more likely to be living in damp homes at 9 months of age, even when taking account of its already disadvantaged socio-economic profile.

Taussig and colleagues (2003) suggested that non-atopic wheezers are probably more likely to develop acute airway obstruction in relation to viral infection because they have an alteration in the control of airway tone that determines this increased risk. Our results could lend support to this hypothesis: trajectory 4 (high wheeze, low levels of other atopic symptoms) appeared to be a disadvantaged group exposed to a number of potential risk factors in infancy, including living in damp homes and not being breastfed. Furthermore, Taussig et al. (2003) concluded that perhaps, for children with atopic symptoms, early (before age 3) allergic sensitization may be an important risk factor for more severe disease. In our results, the risk of living in a damp home at 9 months (which may be linked to allergic sensitization through inhaled mould spores) was highest in two groups with high levels of eczema and/or hayfever.

### Strengths and limitations

The present work enjoys a number of characteristics which increase the robustness of our results. Historically, studies have attempted to classify asthma in an attempt to categorize a complex disease into discrete, clinically useful subsets. In this study, groupings were derived from the observed population heterogeneity using a clustering technique, longitudinal LCA, which requires a relatively small number of subjective choices. LCA clustering is not rigid as each individual is assigned different probabilities of belonging to various classes. This soft form of classification is similar to clinical situations, where patients often present symptoms common to more than one condition. In the present study, the majority of children could be clearly assigned to one class, but for a minority (9%), there remained some ambiguity about the class they were assigned to. Sensitivity analyses using a different classification for these ambiguous cases indicate that the results presented here are robust to this issue.

The second major strength comes from our use of a large, nationally representative and prospective birth cohort study. Our analytical sample included 11,652 children; as a result, each identified trajectory was based on a sample of at least 1,000 children. A second strength of this data is that children are observed from early ages, when the development of lung function, lung responsiveness and atopy is likely to occur. Third, the rich data available in the Millennium Cohort Study means that, rather than just identify trajectories of asthma, we can also attempt to describe them in terms of their socio-economic background, and find early predictors of these trajectories. Significantly, we use data on potentially modifiable factors collected when the child was 9 months old, before diagnoses and treatments of asthma (and, for many children, before even the onset of symptoms) has begun. Therefore, parental report of, for example, problems with damp in the home, might be less likely to be coloured by the child's later symptoms and diagnosis. And we identify predictors when policy intervention could have most impact, as symptoms are just beginning to develop and before the disease is fully entrenched.

Given the nature of the MCS, which tracks a very large number of children and is a multi-disciplinary study, lung function or objective measures of atopy (such as measured by skin prick test or IgE) were not available. Therefore, all outcomes were self-reported, although based on a validated tool. Self-report of symptoms by parents is a widely accepted method in epidemiological studies and reliably reflects the incidence of asthmatic symptoms in young children; furthermore, in preschool children a diagnosis of asthma is often based on symptoms rather clinical measurements. Objective tests, including spirometry or bronchial hyperresponsiveness, are difficult to perform in young children, and especially so within a large cohort of a general population. Furthermore, data on wheeze before 3 years of age was not available.

Wheeze is however a difficult symptom for parents to describe and report. Studies show that parents can have very different perceptions of what wheeze sounds like [44]. While parental-reported wheezing in early life is imprecise [45] and correlates poorly with objective observations [46], previous work found strong correlations between wheezing classifications as derived by parent-reports of wheeze and physician diagnosed asthma at about 7 years [47]. Furthermore, the data used here was collected using the ISAAC module, a widely used and validated questionnaire.

Other atopic symptoms used in our study, such as eczema and hayfever, are also based on parental report. Although symptoms such as eczema have been used as proxy for atopy in previous studies of young children [48], [49], not all children presenting these symptoms are atopic, and some asymptomatic children may be atopic (as identified for example by a skin test or IgE levels). Therefore, some degree of outcome misclassification may be present. However, the prevalence of symptoms in this population-based study was comparable with prevalence rates in equivalent British populations, and we observed consistent associations of our proxy for atopic symptoms with a number of classic variables associated with atopy, such as the child's sex, and the number of older siblings living in the household.

A further limitation to consider is that, as most longitudinal studies, loss to follow-up was greater in children from more socially deprived backgrounds. Given known associations of social deprivation with early childhood wheezing (Baker and Henderson, 1999), it is likely that children excluded because of missing data had higher rates of wheezing than those included in our analyses. As more symptomatic children may not have been included in our analytical sample, it is likely that our estimates are an under representations of the true effects.

Finally, our analytical technique, longitudinal LCA, shares the limitations of other clustering techniques. First, even though researchers use a number of statistical fit criteria as a guide, the problem of determining the number of classes has not been completely resolved. Second, some a priori decisions have to be made, notably about which variables to include in the model. This does introduce a degree of subjectivity to the model. Third, as the term “latent class” implies, these are not directly observed clusters but groups constructed on the basis of the pattern of responses over a fixed number of observation periods. These methods therefore do not predict the natural history of symptoms in an individual, nor produce overall population prevalences, instead trajectories are derived by assigning each child a probability of membership based on their overall symptom history.

## Conclusions

In summary, we use a nationally-representative sample of young British children with longitudinal, prospective information on wheeze and other atopic symptoms and apply latent class techniques to derive trajectories of symptoms over the first seven years of life in a relatively objective and data-driven manner. Four latent trajectories of wheeze and atopy symptoms measured at age 3, 5 and 7 were derived. The rich data set used allowed characterization of the socio-economic profiles of these groups, and studying the association between these latent trajectories to a number of potentially intervenable factors in infancy, before trajectories have entrenched. Exposure to household damp at 9 months, and for a particularly socio-economically disadvantaged group of children with high levels of wheeze, breastfeeding initiation, appeared to be potentially important modifiable factors to focus on.

## Supporting Information

Annex S1 **ISAAC Core Questionnaire for Wheezing and Asthma.**
(DOCX)
