# Supplementary material for: Asthma Trajectories in Early Childhood: Identifying Modifiable Factors
Source: PLoS One. 2014 Nov 7;9(11):e111922. doi: 10.1371/journal.pone.0111922 (PMC4224405; doi:10.1371/journal.pone.0111922)
Supplement: Annex S1 — ISAAC Core Questionnaire for Wheezing and Asthma. (DOCX) [file pone.0111922.s001.docx]

# Annex S1

## ISAAC Core Questionnaire for Wheezing and Asthma

1) Has your child ever had wheezing or whistling in the chest at any time in the past?

2) Has your child had wheezing or whistling in the chest in the last 12 months?

3) How many attacks of wheezing has your child had in the last 12 months?

4) In the last 12 months, how often, on average, has your child's sleep been disturbed due to wheezing?

5) In the last 12 months, has wheezing ever been severe enough to limit your child's speech to only one or two words at a time between breaths?

6) Has your child ever had asthma?

7) In the last 12 months, has your child's chest sounded wheezy during or after exercise?

8) In the last 12 months, has your child had a dry cough at night, apart from a cough associated with a cold or chest infection?
